# Perinatal risks in female cancer survivors: A population-based analysis

**DOI:** 10.1371/journal.pone.0202805

**Published:** 2018-08-23

**Authors:** Anne-Lotte L. F. van der Kooi, David H. Brewster, Rachael Wood, Sian Nowell, Colin Fischbacher, Marry M. van den Heuvel-Eibrink, Joop S. E. Laven, W. Hamish B. Wallace, Richard A. Anderson

**Affiliations:** 1 Department of Obstetrics and Gynecology, Erasmus MC–Sophia Children’s Hospital, Rotterdam, The Netherlands; 2 Princess Máxima Center for Pediatric Oncology, Utrecht, The Netherlands; 3 Scottish Cancer Registry, Information Services Division, NHS National Services Scotland, Edinburgh, Scotland; 4 Information Services Division, NHS National Services Scotland, Edinburgh, Scotland; 5 eData Research & Innovation Service (eDRIS), Information Services Division, NHS National Services Scotland and Farr Institute Scotland, Edinburgh, Scotland; 6 Department of Oncology and Haematology, Royal Hospital for Sick Children, Sciennes Road, Edinburgh, Scotland; 7 MRC Centre for Reproductive Health, University of Edinburgh, Edinburgh, United Kingdom; Monash University, AUSTRALIA

## Abstract

**Background/objectives:**

Advances in cancer management have resulted in improved survival rates, particularly in children and young adults. However, treatment may adversely affect reproductive outcomes among female cancer survivors. The objective of this study was to investigate their risk of adverse perinatal outcomes compared to the general population.

**Design/methods:**

We performed a population-based analysis, including all female cancer survivors diagnosed before the age of 40 years between 1981 and 2012. Pregnancy and perinatal complications were identified through linkage of the Scottish Cancer Registry with hospital discharge records based on the Community Health Index (CHI) database. We compared 1,629 female cancer survivors with a first ever singleton pregnancy after diagnosis, with controls matched on age, deprivation quintile, and year of cancer diagnosis selected from the general population (n = 8,899). Relative risks and 95%-confidence intervals of perinatal risks were calculated using log-binomial regression.

**Results:**

Survivors were more likely to give birth before 37 weeks of gestation (relative risk (RR]) 1.32, 95%-CI 1.10–1.59), but did not show an increased risk of low birth weight (<2.5kg: RR 1.15, 95%-CI 0.94–1.39), and were less likely to give birth to offspring small for gestational age (RR 0.81, 95%-CI 0.68–0.98). Operative delivery and postpartum haemorrhage were more common but approached rates in controls with more recent diagnosis. The risk of congenital abnormalities was not increased (RR 1.01, 95%-CI 0.85–1.20).

**Conclusion:**

Cancer survivors have an increased risk of premature delivery and postpartum haemorrhage, but their offspring are not at increased risk for low birth weight or congenital abnormalities. In recent decades there has been a normalisation of delivery method in cancer survivors, nevertheless careful management remains appropriate particularly for those diagnosed in childhood.

## Introduction

Advances in cancer management have resulted in improved five year survival rates in children and young adults [1]. The impact on later health of survivors is high: quality of life is consistently lower in breast cancer survivors as compared to women without a history of cancer [2, 3], 75% of cancer survivors develop at least one health problem [4], and childhood cancer survivors are 8.2 times more likely to have a severe or life-threatening chronic condition such as premature gonadal failure in comparison to their peers [5, 6]. Fertility is an important issue for survivors [7, 8] but concerns about risks of pregnancy can be a reason to avoid pregnancy [9].

Female survivors of cancer who successfully conceived have been identified to be at risk of premature delivery [10–14] and their offspring have in some studies, but not consistently, been found to be at increased risk of low birth weight [10–13, 15, 16]. Reassuringly, there does not appear to be an increased risk of congenital abnormalities in their offspring [16–22]. Two small studies, one including childhood cancer survivors [23] and one including survivors of cervical cancer treated with cervical conisation [14], did not identify survivors to be at additional risk of caesarean section as mode of delivery. However, in two large population based studies, a British cohort of childhood cancer survivors [10] and a Finnish cohort of survivors of childhood and young adult cancer diagnosed between 0–35 years [24], the rate of elective caesarean section was increased, while the risk of emergency caesarean section was not increased.

The adverse impact of cancer treatment on pregnancy outcomes has to date been investigated in selected patient groups based on diagnosis or age at diagnosis. Reports from the British Childhood Cancer Survivors Study (BCCSS) and the US Childhood Cancer Survivors Study (CCSS) are confined to long-term survivors diagnosed with cancer between 0–14 years from 1940–1991 in Britain (BCCSS) and 0–21 years at diagnosis from 1970–1986 in the 25 participating institutions in Canada and the United States (CCSS) [10, 15, 25, 26]. Other studies excluded the youngest age group and included adolescent and young adult cancer survivors diagnosed with cancer between ages such as 15–39 [11, 20] or 16–45 years [21]. Studies focusing on young adults surviving breast cancer [12, 27], colorectal cancer [28] or cervical cancer [14] have provided insight into perinatal risks in these specific patient groups but their results cannot with confidence be extrapolated to survivors of other types of cancer. Inference of conclusions to current cohorts is limited by the relatively old cohorts often reported. Survivors in both the CCSS and BCSS were diagnosed several decades ago [10, 15, 25, 26], and the treatment regimens administered may no longer be used [8]. Furthermore, reports based on self-reported questionnaires [13, 14, 29, 30] or from specialist paediatric oncology centres such as the CCSS, may under- or overestimate the prevalence of certain events as a result of recall or selection bias, especially when the event was a substantial time ago. Population registries are less prone to recall bias and offer the opportunity to study pregnancy outcomes and perinatal risks at a population level in comparison to the background risk. The objective of this study is to evaluate the perinatal risks among all female survivors from cancer in Scotland diagnosed before 40 years of age in the time period 1981–2012.

## Materials and methods

The Scottish Cancer Registry contains data on cancer diagnoses for all patients in Scotland. All females diagnosed with cancer between 1981 and 2012 before the age of 40 years were identified. They were linked to national general and maternity hospital discharge records to ascertain subsequent first pregnancies leading to delivery of a live, singleton infant up until the end of 2014, using the Community Health Index (CHI) number, a unique identifying number from the CHI database, a population-based register of all patients registered to receive care from the NHS in Scotland. Deliveries occurring less than 6 months following the date of cancer diagnosis were excluded. Population-weighted fifths of Carstairs deprivation scores were assigned to each individual based on census-derived Carstairs scores from 1991 and 2001 for the periods of diagnosis 1981–1995 and 1996–2012, respectively [31]. A comparison group was created from the general population, using the CHI database. For every cancer survivor, three controls were selected matched on age at date of cancer diagnosis/matching and deprivation quintile. Controls had no pregnancies before the date of matching: subsequent first pregnancies leading to delivery of a live singleton infant (at least 6 months after the date of matching) were identified for comparison to deliveries among cancer cases. Only live singleton births in controls and cancer survivors were included in the analyses.

Maternal outcomes that were evaluated included antenatal haemorrhage, postpartum haemorrhage, and mode of delivery: spontaneous vaginal, assisted vaginal, elective caesarean section or emergency caesarean section. Infant outcomes included birthweight, gestational age, small for gestational age (SGA), admission to neonatal unit and congenital abnormalities (ICD codes in S1 Table). Low birthweight was defined as a birthweight <2500 grams, premature delivery as delivery before 37 weeks of gestation and SGA as <10^th^ centile birthweight for gestational age and gender based on the UK90-WHO growth reference [32].

Age at diagnosis of cancer (and its treatment) may affect perinatal risks, therefore data were stratified based on age at diagnosis; 0–14 years; 15–24 years; 25–29 years; 30–34 years; 35–39 years. To evaluate possible effects of socio-economic circumstances, data were stratified based on deprivation fifth. Finally, to investigate possible differences in risk patterns over time, data were stratified into 7-year periods of diagnosis: 1981–1988; 1989–1996; 1997–2004; 2005–2012. P-values for the observed difference were calculated from the two-sample z-test for comparing proportions or t-test for comparing means, and log-binomial regression was employed to calculated risk ratios and 95% confidence intervals. Statistical analyses were conducted in Stata version 14 MP.

The study was approved by the Privacy Advisory Committee of the National Health Service (NHS) National Services Scotland (NSS)–study reference number XRB13215.

## Results

A total of 10,271 nulliparous women diagnosed with cancer before 40 years of age between 1981 and 2012 were identified, of whom 1,629 subsequently delivered a first singleton live birth by end 2014. Of 30,811 nulliparous matched control women, 8,899 delivered a first singleton live birth. The 1,629 survivors and the 8,899 matched control women formed the final cohorts. Half of the cancer survivor cohort had been diagnosed before 25 years of age (48%). The most common malignancies were melanoma and non-melanoma skin cancers (36.7%) followed by Hodgkin lymphoma (11.0%) (Table 1).

**Table 1:** Diagnostic characteristics of 1,669 included female cancer survivors with a subsequent live singleton first ever birth after diagnosis.

	Number	% of included cohort
***Type of first cancer***		
Colorectal	22	1.4
Liver	5	0.3
Bone	27	1.7
Skin (melanoma and NMSC)	598	36.7
Connective and soft tissue	30	1.8
Breast	112	6.9
Cervix uteri	118	7.2
Ovary	105	6.4
Kidney	20	1.2
Eye	8	0.5
Brain, CNS	66	4.1
Thyroid	128	7.9
Hodgkin lymphoma	179	11.0
Non-Hodgkin lymphoma	48	2.9
Leukaemia	81	5.0
Other	82	5.0

NMSC = non-melanoma skin cancers; CNS = central nervous system

Cancer survivors were slightly older at first pregnancy than controls (30.1 vs 28.5 years p<0.001) (Table 1); body mass index (BMI) at booking was similar with 25.5 kg/m^2^, although there were substantial missing data (59.9% in survivors and 72.1% in controls). Smoking was less prevalent in survivors, especially in those diagnosed during childhood (15.1% vs 28.0%, p<0.001) and adolescence (11.0% vs 15.5%, p = 0.005) (Table 1).

Survivors were more likely to deliver prematurely (RR 1.32, 95% CI 1.10–1.59), but did not show a significantly increased risk of low birthweight (RR 1.15, 95% CI 0.94–1.39) (Table 2). Offspring of cancer survivors were less likely to be small for gestational age (RR 0.82, 95% CI 0.68–0.98) than offspring from the general population (Table 3). This difference in gestational adjusted birthweight was not observed in the more recently diagnosed groups or in more deprived quintiles (S2 Table).

**Table 2:** Differences in lifestyle factors between female survivors of cancer and a matched control group.

	Live singleton births (n)		Mean age at 1st pregnancy (years)			Smoking during pregnancy (%)				
	controls	survivors	controls	survivors	p-value^1^	controls		survivors		p-value^2^
						yes	missing	yes	missing	
*Total*	8,899	1,629	28.5	30.1	<0.001	13.7	30.9	10.2	18.0	<0.001
*Age-group at onset of cancer/match (years)*										
0–14	1,292	186	21.2	23.5	<0.001	2.8	16.8	15.1	5.4	<0.001
15–24	2,849	588	24.9	27.2	<0.001	15.5	37.7	11.0	22.4	0.005
25–29	2,367	457	30.0	31.3	<0.001	8.7	33.9	9.2	19.7	0.759
30–34	1,781	306	34.2	35.2	<0.001	8.4	29.8	6.9	15.0	0.376
35–39	610	92	38.4	38.7	0.204	9.2	21.3	10.9	17.4	0.605
*Period of diagnosis of cancer/match*										
1981–1988	2,700	336	26.3	28.3	<0.001	8.1	71.7	7.1	53.9	0.543
1989–1996	2,690	453	27.9	29.1	<0.001	19.5	19.6	11.5	9.1	<0.001
1997–2004	2,063	480	30.2	31.0	0.010	16.3	8.6	12.7	10.2	0.051
2005–2012	1,446	360	31.3	31.6	0.280	9.5	7.7	8.0	6.4	0.376
*Deprivation fifth*										
1 –Least deprived	1,833	328	30.2	31.4	<0.001	7.8	30.6	5.8	14.0	0.199
2	1,684	315	28.8	30.8	<0.001	11.3	30.4	8.3	15.6	0.112
3	1,808	320	28.0	29.9	<0.001	14.4	30.3	8.4	20.0	0.004
4	1,880	356	28.2	29.5	<0.001	15.9	30.5	12.9	18.8	0.153
5 –Most deprived	1,694	310	27.1	28.8	<0.001	19.2	33.0	15.4	21.9	0.118

Female cancer survivors compared to a control group matched on age, diagnosis date and deprivation quintile.

^1^p-value obtained from t-test.

^2^p-value obtained from z-test.

**Table 3:** Relative risk of perinatal outcomes among female survivors of cancer.

	Controls n (%)	Survivors n (%)	RR	LCI	UCI
**Premature birth**	548 (6.2%)	113 (8.2%)	1.32	1.10	1.59
**Low birthweight**	548 (6.2%)	115 (7.1%)	1.15	0.94	1.39
**Small for gestational age**	811 (9.2%)	121 (7.5%)	0.82	0.68	0.98
**Admission to neonatal unit**	1,090 (12.2%)	207 (12.7%)	1.03	0.90	1.19
**Congenital abnormalities**	8,746 (8.4%)	1,593 (9.5%)	1.01	0.85	1.20

Relative risks as compared to a control group matched on age, diagnosis date and deprivation quintile. RR = relative risk; LCI = lower confidence interval; UCI = upper confidence interval. Low birthweight is defined as <2.5 kg; Premature birth is defined as before 37 weeks of gestation; Small for gestational age is defined as under 10^th^ centile for gestational age.

**Fig 1:**
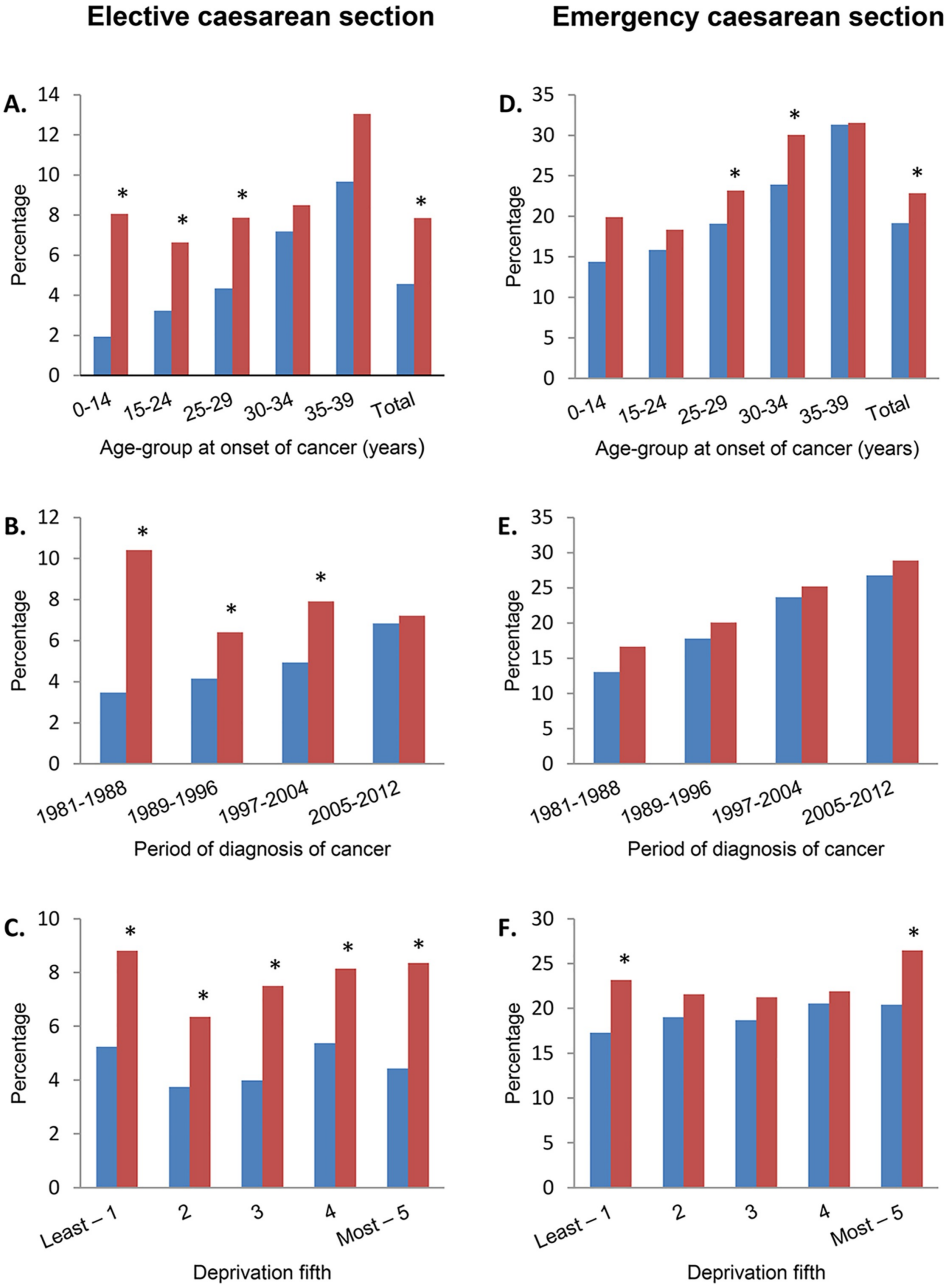
Likelihood of caesarean section in female cancer survivors compared to a matched control group. Panels A, B and C: difference of likelihood on elective caesarean section in female cancer survivors as compared to a matched control group, stratified by age-group at onset of cancer (A), period of diagnosis of cancer (B), and deprivation fifth (C). Panels D, E and F: difference of likelihood on emergency caesarean section by age-group at onset of cancer (D), period of diagnosis of cancer (E), and deprivation fifth (F). Significant differences (p-value < 0.05) between female survivors of cancer and controls are depicted with * per stratified group. Blue bars depict the control group, red bars the cancer survivors.

A spontaneous vaginal delivery was less common in survivors than in the general population (RR 0.72, 95% CI 0.65–0.79) (Table 4). Elective caesarean section was more common in cancer survivors than in the general population (RR 1.59, 95% CI 1.35–1.88), as was emergency caesarean section (RR 1.20, 95% CI 1.08–1.34)). The risk of an elective caesarean section was most increased in women who had been diagnosed aged 0–14 years (RR 3.15, 95% CI 2.04–4.88). There were marked changes by period of diagnosis, with the frequency of operative delivery converging with controls with more recent diagnosis (Fig 1, panel B and E). This was most strikingly seen in the elective caesarean section rate, which declined in cancer survivors while increasing in controls (Fig 1, panel B). In those diagnosed between 1981–1988 the elective caesarean section rates were 10.4% vs 3.5% in controls (p<0.001), while the rates for those diagnosed in 2005–2012 were 7.2% vs 6.8% (p = 0.8) (S3 Table). While in both the survivor and control group the emergency caesarean section rates rose by period of diagnosis, the absolute difference in prevalence remained constant (Fig 1, panel E). Survivors in the lowest and highest quintile of deprivation were more likely to have an emergency caesarean section than their matched peers, while there was no difference in risk for survivors in the middle quintiles (Fig 1, panel F).

**Table 4:** Relative risk of vaginal delivery and haemorrhage among female survivors of cancer.

	Spontaneous vaginal			Assisted vaginal or breech			Antepartum haemorrhage			Postpartum haemorrhage		
	RR	LCI	UCI	RR	LCI	UCI	RR	LCI	UCI	RR	LCI	UCI
Total	0.72	0.65	0.79	1.14	1.00	1.29	1.13	0.86	1.50	1.42	1.29	1.55
*Age-group at onset of cancer (years)*												
0–14	0.63	0.47	0.83	1.25	0.87	1.79	0.55	0.24	1.24	1.62	1.23	2.13
15–24	0.72	0.61	0.84	1.11	0.89	1.39	1.31	0.81	2.13	1.28	1.08	1.53
25–29	0.74	0.62	0.89	1.12	0.88	1.41	1.47	0.86	2.49	1.65	1.40	1.96
30–34	0.65	0.52	0.82	1.25	0.94	1.65	1.35	0.69	2.66	1.33	1.09	1.61
35–39	0.87	0.58	1.32	0.98	0.56	1.70	1.21	0.43	3.42	1.30	0.92	1.83
*Period of diagnosis of cancer*												
1981–1988	0.56	0.45	0.69	1.07	0.76	1.48	0.73	0.23	2.37	1.70	1.29	2.23
1989–1996	0.72	0.60	0.87	1.09	0.82	1.45	0.87	0.50	1.50	1.31	1.07	1.61
1997–2004	0.88	0.74	1.04	1.04	0.83	1.29	0.91	0.57	1.47	1.24	1.06	1.45
2005–2012	0.89	0.74	1.08	1.04	0.84	1.30	1.54	0.93	2.55	1.16	1.00	1.35
*Deprivation fifth*												
1 –Least deprived	0.65	0.53	0.80	1.10	0.84	1.45	1.40	0.79	2.48	1.62	1.36	1.93
2	0.74	0.60	0.92	1.13	0.84	1.50	0.85	0.39	1.87	1.34	1.08	1.67
3	0.76	0.61	0.94	1.07	0.79	1.44	1.34	0.74	2.42	1.30	1.03	1.64
4	0.80	0.65	0.97	1.11	0.85	1.46	0.81	0.40	1.61	1.45	1.18	1.78
5 –Most deprived	0.65	0.52	0.80	1.33	1.00	1.76	1.25	0.71	2.21	1.33	1.06	1.67

Relative risks as compared to a control group matched on age, diagnosis date and deprivation quintile. RR = relative risk; LCI = lower confidence interval; UCI = upper confidence interval.

There was no marked increased risk of antepartum haemorrhage (RR 1.13, 95% CI 0.86–1.50) for the cancer survivors. Postpartum haemorrhage occurred more often in cancer survivors (RR 1.42, 95% CI 1.29–1.55) (Table 4). The prevalence of postpartum haemorrhage increased in the control general population over time from 9.3% to 33.1%, while in the cancer survivors the prevalence increased from 15.8% to 38.3% over time with the prevalence of postpartum haemorrhage being similar to the control general population for later treated cohorts. (S4 Table). The risk of postpartum haemorrhage was most increased in women who had been diagnosed aged 0–14 years (RR 1.62, 95% CI 1.23–2.13), while no increased risk was observed in women diagnosed between 35–39 years (RR 1.30, 95% CI 0.92–1.83).

Offspring of cancer survivors were equally likely to be admitted to a neonatal unit (RR 1.03, 95% CI 0.90–1.19) and showed no increased risk of congenital abnormalities (RR 1.01, 95% CI 0.85–1.20) (Table 3).

## Discussion

### Main findings

This population-based study compared the frequency of adverse perinatal outcomes in cancer survivors diagnosed in Scotland before 40 years of age between 1981–2012 and non-cancer controls matched from the general population. Survivors were more at risk of a preterm delivery but their offspring were not at increased risk of low birthweight and had a decreased risk of SGA. Elective caesarean section was more common in cancer survivors as was emergency caesarean section, but there were marked changes by period of diagnosis, with the frequency of both elective and emergency caesarean section converging with controls among those with a more recent diagnosis. Similar findings of increased but converging risk were found for postpartum haemorrhage. The risk of congenital abnormalities in offspring of cancer survivors was not increased.

### Strengths and limitations

The major strengths of this study include the population-based approach using national registry data, which allowed evaluation of all first singleton pregnancy outcomes in female survivors from cancer, diagnosed at an age under 40 years. A large age matched non-cancer control group was identified from the general population. Pregnancy outcomes were accurately recorded and free of recall bias, but this study lacks cancer treatment information including radiotherapy to the abdomen and pelvis as this is not routinely collected in the databases used for this study. We report on the perinatal risks of female survivors from all cancers, which results in a heterogeneous cohort with regard to their diagnosis and treatment. The most common malignancies were melanoma and non-melanoma skin cancers which are more commonly treated with local therapy with lesser likelihood to impact future perinatal risks. Although all presented relative risks are compared to an age and period matched control group, the follow-up for patients diagnosed in the most recent period of diagnosis is still relatively short, especially for childhood cancer survivors. This may have influenced the observed trends.

### Interpretation

Cancer survivors achieve fewer pregnancies in comparison to the general population, with an overall reduction in likelihood of pregnancy after diagnosis of 38% [33]. Concerns about risks of pregnancy are sufficient reason to avoid pregnancy for some survivors [9]. Overall, our results are reassuring to cancer survivors who wish to become pregnant. We observed no increased risk of congenital abnormalities, which is consistent with previous studies of risk of congenital malformations in offspring of cancer survivors, which also found no an associations with radiotherapy or chemotherapy treatment [16–22, 34]. Our results of increased risk of premature delivery among cancer survivors agree with previously reported studies [10–14]. This has been particularly linked to radiotherapy to fields which include the uterus, particularly in pre-pubertal girls [35], which can lead to reduced uterine volume and elasticity [36, 37]. In addition, uterine vascularisation may be impaired, with potential detrimental consequences for fetal-placental blood flow causing fetal growth restrictions. Our results of no increased risk of low birthweight are consistent with earlier findings in cohorts with survivors from cancer at a young [10, 13] and adult [38] age, although in the BCCSS cohort there was an increased risk in the subgroup that received radiation to a field that included the abdomen [10]. Other reports in cohorts with women diagnosed at a young age however, did show an increased risk of low birthweight [16], as did cohorts of women surviving breast cancer [12] and women diagnosed aged 15–39 years [11]. Maternal smoking is a well-recognised risk factor for low birthweight [39] and having a small for gestational age baby [40]. In our study, cancer survivors were less likely to smoke during pregnancy than the control general population, especially those diagnosed in childhood and adolescence. As the prevalence of smoking decreased in the general population in later periods, the differences between cancer survivors and the control general population converged. This may suggest that cancer survivors were more aware of the harmful risks of smoking than the control general population and more inclined to stop or not to start smoking. This supports the value of ongoing health surveillance in this group [41]. The z-score of mean birthweight also converged by period of diagnosis, illustrating that offspring of cancer survivors diagnosed in the eighties and nineties had a higher birthweight than their control peers, a difference that diminished in the offspring of survivors diagnosed after 1997. The lower prevalence of smoking during pregnancy in cancer survivors in our study population may have in part counteracted the negative effects that treatment strategies such as uterine radiation have on uterine elasticity. Their earlier adoption of a healthier lifestyle may have been beneficial to the risk of delivery of offspring that were small for gestational age. Unfortunately, information on smoking during pregnancy was missing in a substantial proportion of the cancer survivors, and in an even larger proportion of the control general population. These non-randomly missing data prohibited adjustment for this possible confounder, as excluding those with missing data from the analysis may lead to biased results [42]. Offspring of the least deprived survivors also had slightly higher birthweights than their matched controls.

Previous studies have indicated that cancer survivors are at increased risk of postpartum haemorrhage, but only after abdominal radiation [10, 23] although other studies have reported no increased risk [20, 24, 43]. We show a higher risk of postpartum haemorrhage in cancer survivors overall, but as the incidence of postpartum haemorrhage has increased more rapidly in the control general population, the difference has diminished in the most recent decade. It is possible that better recording of haemorrhage in routine records (reporting bias), as a result of intensified surveillance, may have played a role in the higher reported incidence of postpartum haemorrhage in cancer survivors.

A lower threshold for intervention may at least in part explain the higher incidence of both emergency and elective caesarean section in the cancer survivors, although rates of elective section in particular converged with controls by period of diagnosis. The rate of emergency caesarean section rose by period of diagnosis in both the survivor and control groups, and there was no significant difference in risk for any single period of diagnosis. The over three-fold increased risk of an elective caesarean section in the women diagnosed aged 0–14 is substantially larger than the impact observed (RR 1.38 in those not treated with radiotherapy and RR 1.46 in those treated with abdominal radiotherapy) in the BCCSS [10]. In that study 40% of survivors were diagnosed in the most recent time period included (1985–1991), with pregnancy outcomes between 1997 and 2012, thus the pregnancy outcomes may be more comparable to the more recently diagnosed (and more recently pregnant) cohorts in the present data.

Inequalities by deprivation were found in the prevalence of operative delivery, where only the most and least deprived show an increased risk of emergency caesarean section, whereas survivors in all deprivation quintiles were at increased risk of elective caesarean section. Deprivation is known to be a major factor in increasing health inequalities [44]. It is possible that survivors from the most deprived group may experience greater medical intervention in their obstetric care due to the presence of more co-morbidities whereas less deprived survivors may be more empowered to influence their obstetric care. However these differences require specific investigation to confirm and determine their basis.

As with the normalized risk of postpartum haemorrhage over time, the impact of a cancer diagnosis on the risk of an operative delivery also diminished in the later periods, resulting in equal risks of all modes of delivery for those diagnosed in the most recent cohort. The reduced impact of a cancer diagnosis on the risk of an intervention during delivery may be a result of better targeted treatment strategies, and of a reduction of therapeutic exposures known to be associated with organ toxicity, e.g. radiotherapy in Hodgkin lymphoma [45]. This observation is also in line with decreased late mortality among survivors of childhood cancer as a result of reduced radiotherapy and chemotherapy exposure [46].

## Conclusion

Cancer survivors are at increased risk of premature delivery and postpartum haemorrhage, but not of small for gestational age or congenital abnormalities when compared to a non-cancer control population. It is reassuring that the impact of a cancer diagnosis on postpartum haemorrhage and mode of delivery has been greatly reduced in most recently diagnosed cohorts of survivors, although heightened alertness and careful management in cancer survivors remains appropriate.

## Supporting information

S1 Table International Statistical Classification of Disease and Related Health Problems (ICD) codes used to identify included cancers and perinatal outcomes. International Statistical Classification of Disease and Related Health Problems (ICD) codes used to identify included cancers and perinatal outcomes.
(DOCX)

S2 Table Mean birthweight z-scores. Female cancer survivors compared to a control group matched on age, diagnosis date and deprivation quintile. ^1^p-value obtained from t-test.
(DOCX)

S3 Table Frequencies of modes of delivery in controls and cancer survivors. Frequencies of modes of delivery in controls and cancer survivors.
(DOCX)

S4 Table Frequencies of antepartum haemorrhage and postpartum haemorrhage in controls and cancer survivors. Frequencies of antepartum haemorrhage and postpartum haemorrhage in controls and cancer survivors.
(DOCX)
