# Supplementary material for: Perinatal risks in female cancer survivors: A population-based analysis
Source: PLoS One. 2018 Aug 23;13(8):e0202805. doi: 10.1371/journal.pone.0202805 (PMC6107257; doi:10.1371/journal.pone.0202805)
Supplement: S1 Table — International Statistical Classification of Disease and Related Health Problems (ICD) codes used to identify included cancers and perinatal outcomes. (DOCX) [file pone.0202805.s001.docx]

**S1 Table.**  International Statistical Classification of Disease and Related Health Problems (ICD) codes used to identify included cancers and perinatal outcomes

| **Cancer** | **ICD10 Codes*** |
| --- | --- |
| Colorectal | C18-C20 |
| Liver | C22 |
| Bone | C40-C41 |
| Skin (melanoma and NMSC) | C43, C44 |
| Connective and soft tissue | C47, C49 |
| Breast | C50 |
| Cervix uteri | C53 |
| Ovary | C56 |
| Kidney | C64 |
| Eye | C69 |
| Brain, CNS | C70-C72, C75.1-C75.3 |
| Thyroid | C73 |
| Hodgkin lymphoma | C81 |
| Non-Hodgkin lymphoma | C82-C85, C96 |
| Leukaemia | C90.1, C91-C95 |
| Other | All other codes in the range C00-C96 |
| All combined | C00-C96 |

*Records entered before the introduction of ICD-10 have been forward mapped, using the combination of ICD-9 code and morphology codes.

Date Ranges

- ICD-9 – 1981 to March 1996 ICD-10 – April 1996 to present
- OPCS3 – 1977 to 1988 OPCS4 – 1989 to present

|  | **ICD-9** | **ICD-10** |
| --- | --- | --- |
| **Perinatal outcome** |  |  |
| Congenital Anomalies | 216,228,605, 4253, 550-553, 740-759 | D18, D22-D23, I424, K40-K46, all Q codes |
| Postpartum haemorrhage | 666 | O72 |
| Antepartum haemorrhage | 6413, 6418, 6419, 6412, 6441 | O441,O45, O46 |

Congenital Abnormalities were identified using ICD codes recorded in Neonatal datasets from 1981 to 2002, Scottish Birth Records from 2003 onwards and hospital discharge records and Infant deaths from 1981 to 2014. Only records that occurred in the first 12 months of the child’s life were used.
