# Supplementary material for: Perinatal risks in female cancer survivors: A population-based analysis
Source: PLoS One. 2018 Aug 23;13(8):e0202805. doi: 10.1371/journal.pone.0202805 (PMC6107257; doi:10.1371/journal.pone.0202805)
Supplement: S2 Table — Female cancer survivors compared to a control group matched on age, diagnosis date and deprivation quintile. 1p-value obtained from t-test. (DOCX) [file pone.0202805.s002.docx]

**S2 Table.** Mean birthweight z-scores

|  | **mean birthweight z score** | | |
| --- | --- | --- | --- |
|  | **Controls** | **Survivors** | **p-value^1^** |
| Total | -0.0597 | 0.0651 | <0.001 |
|  |  |  |  |
| *Age-group at onset of cancer/match (years)* |  |  |  |
| 0-14 | -0.1023 | -0.0983 | 0.95 |
| 15-24 | -0.1110 | 0.1029 | <0.001 |
| 25-29 | -0.0143 | 0.1258 | 0.01 |
| 30-34 | -0.0331 | 0.0028 | 0.56 |
| 35-39 | 0.0148 | 0.0589 | 0.70 |
|  |  |  |  |
| *Period of diagnosis of cancer/match* |  |  |  |
| 1981-1988 | -0.1307 | 0.0846 | <0.001 |
| 1989-1996 | -0.0754 | 0.0983 | <0.001 |
| 1997-2004 | -0.0337 | 0.0259 | 0.23 |
| 2005-2012 | 0.0645 | 0.0575 | 0.90 |
|  |  |  |  |
| *Deprivation fifth* |  |  |  |
| 1 – Least deprived | -0.0111 | 0.1546 | <0.001 |
| 2 | -0.0028 | 0.1017 | 0.08 |
| 3 | -0.0443 | 0.0972 | 0.02 |
| 4 | -0.0765 | -0.0459 | 0.60 |
| 5 – Most deprived | -0.1667 | 0.0279 | 0.19 |

Female cancer survivors compared to a control group matched on age, diagnosis date and deprivation quintile. ^1^p-value obtained from t-test.
