# Supplementary material for: Perinatal risks in female cancer survivors: A population-based analysis
Source: PLoS One. 2018 Aug 23;13(8):e0202805. doi: 10.1371/journal.pone.0202805 (PMC6107257; doi:10.1371/journal.pone.0202805)
Supplement: S3 Table — Frequencies of modes of delivery in controls and cancer survivors. (DOCX) [file pone.0202805.s003.docx]

**S3 Table.** Frequencies of modes of delivery in controls and cancer survivors

|  | **spontaneous vaginal** | | | **assisted vaginal or breech** | | | **elective caesarean section** | | | **emergency caesarean section** | | | **unknown delivery mode** | |
| --- | --- | --- | --- | --- | --- | --- | --- | --- | --- | --- | --- | --- | --- | --- |
|  | **controls** | **cancer survivors** | **p-value** | **controls** | **cancer survivors** | **p-value** | **controls** | **cancer survivors** | **p-value** | **controls** | **cancer survivors** | **p-value** | **controls** | **cancer survivors** |
| Total | 53.8% | 45.0% | <0.001 | 12.8% | 14.7% | 0.0446 | 4.6% | 7.9% | <0.001 | 19.2% | 22.8% | 0.001 | 9.6% | 9.6% |
|  |  |  |  |  |  |  |  |  |  |  |  |  |  |  |
| *Age-group at onset of cancer/match (years)* | |  |  |  |  |  |  |  |  |  |  |  |  |  |
| 0-14 | 65.1% | 53.2% | 0.002 | 13.0% | 16.1% | 0.2419 | 1.9% | 8.1% | <0.001 | 14.4% | 19.9% | 0.050 | 5.6% | 2.7% |
| 15-24 | 60.0% | 51.4% | <0.001 | 11.5% | 12.9% | 0.3330 | 3.2% | 6.6% | <0.001 | 15.9% | 18.4% | 0.135 | 9.4% | 10.7% |
| 25-29 | 50.7% | 42.9% | 0.002 | 13.7% | 15.3% | 0.3706 | 4.4% | 7.9% | 0.001 | 19.1% | 23.2% | 0.044 | 12.1% | 10.7% |
| 30-34 | 45.3% | 34.3% | <0.001 | 13.1% | 16.3% | 0.1314 | 7.2% | 8.5% | 0.418 | 23.9% | 30.1% | 0.021 | 10.5% | 10.8% |
| 35-39 | 37.0% | 33.7% | 0.534 | 14.4% | 14.1% | 0.9399 | 9.7% | 13.0% | 0.317 | 31.3% | 31.5% | 0.968 | 7.5% | 7.6% |
|  |  |  |  |  |  |  |  |  |  |  |  |  |  |  |
| *Period of diagnosis of cancer/match* | |  |  |  |  |  |  |  |  |  |  |  |  |  |
| 1981-1988 | 62.2% | 48.5% | <0.001 | 9.8% | 10.4% | 0.7109 | 3.5% | 10.4% | <0.001 | 13.0% | 16.7% | 0.066 | 11.5% | 14.0% |
| 1989-1996 | 55.8% | 47.9% | 0.002 | 9.1% | 9.9% | 0.5564 | 4.2% | 6.4% | 0.033 | 17.8% | 20.1% | 0.244 | 13.2% | 15.7% |
| 1997-2004 | 47.1% | 43.3% | 0.140 | 15.5% | 16.0% | 0.7528 | 4.9% | 7.9% | 0.010 | 23.7% | 25.2% | 0.487 | 8.8% | 7.5% |
| 2005-2012 | 43.8% | 40.3% | 0.230 | 21.9% | 22.8% | 0.7050 | 6.8% | 7.2% | 0.802 | 26.8% | 28.9% | 0.417 | 0.8% | 0.8% |
|  |  |  |  |  |  |  |  |  |  |  |  |  |  |  |
| *Deprivation fifth* | |  |  |  |  |  |  |  |  |  |  |  |  |  |
| 1 – Least deprived | 51.9% | 40.5% | <0.001 | 14.9% | 16.5% | 0.4812 | 5.2% | 8.8% | 0.010 | 17.3% | 23.2% | 0.011 | 10.6% | 11.0% |
| 2 | 55.2% | 47.3% | 0.010 | 12.9% | 14.6% | 0.4251 | 3.7% | 6.3% | 0.033 | 19.0% | 21.6% | 0.287 | 9.1% | 10.2% |
| 3 | 56.6% | 49.4% | 0.017 | 12.0% | 12.8% | 0.6823 | 4.0% | 7.5% | 0.005 | 18.7% | 21.3% | 0.284 | 8.7% | 9.1% |
| 4 | 52.8% | 46.6% | 0.032 | 12.9% | 14.3% | 0.4561 | 5.4% | 8.1% | 0.040 | 20.5% | 21.9% | 0.557 | 8.4% | 9.0% |
| 5 – Most deprived | 52.4% | 41.0% | <0.001 | 11.3% | 15.2% | 0.0559 | 4.4% | 8.4% | 0.003 | 20.4% | 26.5% | 0.017 | 11.5% | 9.0% |

Female cancer survivors compared to a control group matched on age, diagnosis date and deprivation quintile. P-values obtained from t-test.
