# Supplementary material for: Perinatal risks in female cancer survivors: A population-based analysis
Source: PLoS One. 2018 Aug 23;13(8):e0202805. doi: 10.1371/journal.pone.0202805 (PMC6107257; doi:10.1371/journal.pone.0202805)
Supplement: S4 Table — Frequencies of antepartum haemorrhage and postpartum haemorrhage in controls and cancer survivors. (DOCX) [file pone.0202805.s004.docx]

**S4 Table.** Frequencies of antepartum haemorrhage and postpartum haemorrhage in controls and cancer survivors

|  | **Antepartum haemorrhage** | | | **Postpartum haemorrhage** | | |
| --- | --- | --- | --- | --- | --- | --- |
|  | **controls** | **cancer survivors** | **p-value** | **controls** | **cancer survivors** | **p-value** |
| Total | 3.1% | 3.5% | 0.385 | 18.3% | 25.9% | <0.001 |
|  |  |  |  |  |  |  |
| *Age-group at onset of cancer/match (years)* |  |  |  |  |  |  |
| 0-14 | 5.9% | 3.2% | 0.139 | 15.9% | 25.8% | 0.001 |
| 15-24 | 2.6% | 3.4% | 0.276 | 16.7% | 21.4% | 0.006 |
| 25-29 | 2.5% | 3.7% | 0.154 | 17.2% | 28.4% | 0.000 |
| 30-34 | 2.4% | 3.3% | 0.381 | 22.1% | 29.4% | 0.005 |
| 35-39 | 3.6% | 4.3% | 0.726 | 23.4% | 30.4% | 0.145 |
|  |  |  |  |  |  |  |
| *Period of diagnosis of cancer/match* |  |  |  |  |  |  |
| 1981-1988 | 1.2% | 0.9% | 0.599 | 9.3% | 15.8% | 0.000 |
| 1989-1996 | 3.6% | 3.1% | 0.608 | 15.2% | 19.9% | 0.011 |
| 1997-2004 | 4.6% | 4.2% | 0.710 | 23.7% | 29.4% | 0.010 |
| 2005-2012 | 3.6% | 5.6% | 0.089 | 33.1% | 38.3% | 0.059 |
|  |  |  |  |  |  |  |
| *Deprivation fifth* |  |  |  |  |  |  |
| 1 – Least deprived | 3.1% | 4.3% | 0.253 | 21.3% | 34.5% | <0.001 |
| 2 | 2.6% | 2.2% | 0.687 | 18.5% | 24.8% | 0.010 |
| 3 | 3.0% | 4.1% | 0.339 | 16.8% | 21.9% | 0.028 |
| 4 | 3.1% | 2.5% | 0.539 | 17.3% | 25.0% | 0.001 |
| 5 – Most deprived | 3.6% | 4.5% | 0.435 | 17.5% | 23.2% | 0.016 |

Female cancer survivors compared to a control group matched on age, diagnosis date and deprivation quintile. P-values obtained from t-test.
